# Spatiotemporal properties in cognitive map formation: context-dependent effects of landmarks and perspective

**DOI:** 10.1186/s41235-026-00751-9

**Published:** 2026-08-18

**Authors:** Lei Huang, Junheng Zhang, Kuo Meng, Kuiliang Li, Jing Wang, Ming Ji

**Affiliations:** 1 School of Psychology, Shaanxi Normal University, Xi’an, 710062 Shaanxi China; 2 Shaanxi Key Laboratory of Behavior and Cognitive Neuroscience, No.199, South Chang’an Road, Yanta District, Xi’an, 710062 Shaanxi China

**Keywords:** Cognitive map, Spatiotemporal properties of navigation, Landmarks, Spatial reference frames, Navigation

## Abstract

Effective navigation depends on the formation of cognitive maps, yet it remains unclear how temporal experience is integrated with spatial information during this process. While prior research has primarily emphasized external spatial cues, real-world navigation unfolds over time, raising the possibility that cognitive maps emerge from the integration of temporal and spatial information. The present study examined how the spatiotemporal properties of navigation—operationalized as navigation-duration—interacted with landmark availability and navigational perspective to shape different forms of spatial knowledge. A total of 149 participants completed a virtual navigation task, followed by measures of path distance estimation, landmark sequence memory, and sketch mapping. Results showed that navigation-duration selectively biased metric distance judgments but did not affect landmark sequence memory or sketch-map accuracy, indicating a dissociation between metric and structural representations. Critically, the effects of temporal information were modulated by environmental and representational context: aerial perspective improved layout accuracy only in the presence of landmarks, whereas ground-level perspective yielded more stable performance in their absence. Regression analyses further revealed that landmark knowledge, perspective, spatiotemporal properties, and sense of direction independently contributed to cognitive map accuracy. Together, these findings support a context-dependent integration account of cognitive map formation, in which temporal and spatial information are flexibly weighted depending on environmental structure and representational format. This work provides a mechanistic explanation for systematic spatial distortions and highlights the role of the spatiotemporal properties of navigation in cognitive map construction.

## Significance Statement

Everyday navigation—whether walking through unfamiliar buildings, driving in complex road networks, or following GPS-based navigation systems—depends on how people form internal maps of space. Yet these cognitive maps are often systematically distorted, particularly in dynamic, time-constrained environments such as hospitals, airports, or urban traffic systems. Traditional approaches to improving navigation have focused primarily on optimizing spatial information, such as clearer signage, more salient landmarks, or more precise digital maps. However, real-world navigation is inherently a spatiotemporal process: individuals continuously integrate where they are with how long they have been moving, waiting, or making decisions. This raises a critical and practical question: how does temporal experience shape spatial understanding in real-world navigation contexts? The present research addresses this question by examining how temporal experience, environmental cues, and perspective jointly influence the formation of cognitive maps. Using controlled virtual environments, the study demonstrates that time is not merely an accompanying factor but rather biases spatial judgments, particularly distance estimation, and that its influence depends on the availability of landmarks and the format of spatial representation. These findings suggest that navigation errors—whether occurring during wayfinding, driving, or GPS use—may arise not only from insufficient spatial information but also from how temporal and spatial information are integrated. By identifying the conditions under which such distortions emerge, this work provides a foundation for improving the design of navigation systems, including digital mapping tools, in-vehicle guidance interfaces, and spatial training programs in complex environments.

## Introduction

Spatial navigation is a fundamental ability that supports everyday functioning, occupational performance, and adaptive interaction with complex environments. However, even individuals with relatively strong navigational abilities may experience disorientation and spatial orientation deficits when confronted with environmental changes (Kelly et al., 2008; Krzyś et al., 2026). One possible explanation for these difficulties may lie in individual differences in the construction of cognitive maps. Cognitive maps are generally understood as internal representations of environmental structure that support flexible route selection and spatial inference (Park et al., 2020; Wolbers & Hegarty, 2010). Critically, a cognitive map is not a veridical copy of the external environment but a constructed representation that varies across individuals in precision, coherence, and structural integration (Ho et al., 2022). Consistent with this view, previous research has shown that navigators may overestimate complex path lengths during navigation and exhibit directional distortions relative to objective layouts (Meilinger et al., 2018; Moar & Bower, 1983; Peer et al., 2021).

While these distortions are typically interpreted as arising from limitations in spatial encoding or integration (Epstein et al., 2017), such accounts implicitly assume that cognitive map formation is driven primarily by spatial information alone (Mou, 2025). This assumption leaves open a critical question: whether additional cognitive processes might systematically shape or bias the construction of spatial representations. Research in spatial cognition has traditionally emphasized the encoding of external spatial information—such as distances, directions, and landmarks—treating navigation primarily as a problem of spatial structure (Ishikawa & Zhou, 2020; Kozhevnikov & Puri, 2023). However, this emphasis overlooks a fundamental property of real-world navigation: it unfolds over time, often under conditions of environmental uncertainty, temporal constraints, and dynamically changing contextual demands. Accordingly, navigation may involve not only the processing of spatial structure but also the integration of temporal experience during movement through the environment (Epstein et al., 2017). From this perspective, the spatiotemporal properties of navigation may constitute an important dimension of cognitive map formation. Teghil et al. (2019) proposed that these properties may be reflected in the processing of sequential landmark information along routes and in the estimation of path lengths connecting environmental locations. Converging evidence further suggests that variations in navigation-duration may influence judgments of path length, route complexity, and perceived spatial scale, indicating that temporal information may actively contribute to the construction of spatial representations rather than merely accompany them (Bratzke et al., 2024; Jagorska & Riemer, 2024). These findings raise the possibility that spatial knowledge may not be constructed solely from external spatial cues, but also constrained by internal temporal processing. Therefore, the present research does not treat temporal processing as independent from spatial cognition. Instead, it adopts an integrative perspective that focuses on the joint contribution of temporal and spatial information to cognitive map formation (Loeffler et al., 2018). More specifically, the present research examines whether the spatiotemporal properties of navigation influence the formation and organization of cognitive maps during spatial navigation. This issue remains insufficiently understood, despite the fact that cognitive map formation requires the integration of multiple sources of information across extended navigational experiences (Teghil et al., 2019). Understanding how temporal processing interacts with external spatial information may therefore provide a more mechanistic account of the distortions and variability commonly observed in cognitive map construction.

### Spatiotemporal properties of navigation and cognitive map formation

The spatiotemporal properties of navigation may be reflected in individuals’ tendency to integrate temporal information with spatial events during navigation, resulting in a continuously updated representation of the environment that includes the encoding of sequential landmark information encountered along routes as well as the estimation of path lengths connecting identical landmarks (Teghil et al., 2019). This spatiotemporal coupling is evident not only in cognition but also in language use. Spatial distance is frequently expressed in temporal terms (e.g., “a ten-minute walk”), whereas temporal experience is frequently structured using spatial metaphors (e.g., “moving forward in time”). Such patterns may suggest that spatial and temporal information share partially overlapping representational systems (Boroditsky, 2000). Empirical evidence further indicates that spatial and temporal processing may influence one another bidirectionally. Increases in spatial distance or path complexity reliably prolong subjective navigation time (Riemer et al., 2018), whereas compression or expansion of travel time alters judgments of distance and environmental scale (Huang et al., 2024; Kaski et al., 2016; Teghil et al., 2019). Moreover, environmental features such as turn frequency, obstacles, and landmark transitions disrupt temporal experience, thereby introducing systematic distortions into spatial representations (Brunec et al., 2017, 2020). Previous studies have suggested that increased navigational exposure may facilitate the acquisition and integration of spatial metric information, potentially improving spatial judgments under some conditions (Thorndyke & Hayes-Roth, 1982). However, other studies indicate that prolonged navigation-duration may simultaneously introduce temporal distortion-related biases into distance estimation (Brunec et al., 2017). Neuroimaging evidence further suggests that spatial imagery and temporal reconstruction recruit partially overlapping neural regions, including the right inferior parietal cortex, implying that spatial and temporal processing may rely on shared neural mechanisms (Gauthier & van Wassenhove, 2016). These findings indicate that spatiotemporal properties of navigation may constitute an intrinsic dimension of cognitive map formation. Variations in spatiotemporal processing may influence judgments of environmental structure and the precision of spatial memory. Nevertheless, a critical question remains unresolved: does the influence of spatiotemporal properties of navigation depend on environmental cues or reference frames? Accordingly, one of the primary aims of the present study is to elucidate the role of the spatiotemporal properties of navigation in cognitive map formation.

### The functional role of landmarks in the spatiotemporal properties of navigation

Spatial knowledge formation depends not only on continuous bodily movement cues but also on environmental information available within the navigational context (Mao & Gu, 2025). Among these cues, landmarks—stable and perceptually salient features of the environment—may play an important role in spatial cognition by supporting directional inference, place recognition, and memory for route sequences (Wang et al., 2014). Landmarks appear to serve two related functions during navigation. First, they may act as reference anchors that facilitate the encoding of positional relations and environmental structure (Wunderlich & Gramann, 2021). Second, they may function as salient events along navigational routes, segmenting continuous experience into discrete units and thereby supporting the sequential encoding of route knowledge and topological relations among path segments (Peer et al., 2021). Crucially, these spatial functions of landmarks are also reflected in the spatiotemporal properties of spatial navigation. Temporal experience is known to be influenced by the density of events perceived within a given interval (Jovanovic & Mamassian, 2019). Accordingly, landmarks may influence spatial representation not only through spatial reference information but also by influencing the temporal structure through which navigational experience is encoded.

Importantly, the role of landmarks in the spatiotemporal processing of navigation may depend on navigational context (Bicanski & Burgess, 2020; He et al., 2021; Shin & DuBrow, 2021). In route-based navigation, landmarks may primarily function as sequential events that structure continuous experience into ordered segments (Shin & DuBrow, 2021). In contrast, when the global layout of the environment is visually accessible, landmarks may more strongly support metric and directional coding by serving as stable spatial reference points (Wunderlich & Gramann, 2021). These findings suggest that landmarks may influence cognitive map formation through different mechanisms depending on the available spatial context and reference frame.

### Spatial reference frames and the spatiotemporal properties of navigation

The role of the spatiotemporal properties of navigation and landmarks in cognitive map formation further raises the question of whether these effects remain stable across different navigational contexts. One factor that may shape such variation is the spatial reference frame, which influences how spatial information is selected, organized, and encoded. During navigation, individuals typically rely on cues such as optic flow and environmental changes to estimate navigation-duration (Brunec et al., 2017, 2020). Because these cues may be processed differently across reference frames, the contribution of temporal information to spatial judgments may also vary as a function of the reference frame employed (Ekstrom & Ranganath, 2018). Different spatial reference frames provide distinct coordinate systems for encoding and updating navigational information (Bicanski & Burgess, 2020). Egocentric reference frames rely primarily on self-motion cues and body-centered coordinates, whereas allocentric reference frames emphasize stable relations within the environment (Li & Abarbanell, 2019; Török et al., 2014). To examine how the spatiotemporal properties of navigation contribute to spatial representation, the present study manipulated navigational perspective as a means of engaging different spatial reference frames (Török et al., 2014). A ground-level perspective situates individuals as active agents moving through space, naturally engaging an egocentric reference frame. Under this perspective, spatial information is encoded along the unfolding trajectory of movement, and the encoding of spatial locations may be influenced by the temporal order in which objects are encountered, suggesting a coupling between spatial input and temporal sequential cues (Iachini et al., 2023; Török et al., 2014). Landmark transitions may consequently be processed as discrete navigational events (Shin & DuBrow, 2021). At the same time, the perceived timing of an event may be biased by temporally adjacent events (Jovanovic & Mamassian, 2020), potentially influencing judgments of navigation-duration and related spatial estimates. In contrast, an aerial perspective emphasizes the global configuration of the environment while attenuating bodily and motion-related cues, thereby promoting an allocentric reference frame (Török et al., 2014). Under this perspective, spatial judgments may rely more heavily on stable structural relations within the environment, reducing dependence on local events (Iachini et al., 2023). Taken together, these findings suggest that spatial reference frames influence how temporal and spatial information are integrated, thereby giving rise to distinct patterns of bias in spatial judgment.

Building on this framework, the present study manipulates ground versus aerial perspectives to activate distinct spatial reference frames and examines how landmark availability and spatiotemporal properties of navigation differentially shape spatial representations across these contexts.

### Research aims and hypotheses

The present study examined whether the influence of the spatiotemporal properties of navigation on different forms of spatial knowledge in cognitive maps varies as a function of environmental structure and navigational perspective. Specifically, we investigated whether navigation-duration, landmark availability, and perspective differentially affect multiple forms of spatial knowledge, including path distance estimation, landmark sequence memory, and sketch mapping.

#### H1

Navigation-duration is expected to influence the processing of metric spatial knowledge (Teghil et al., 2019). Previous literature suggests two competing possibilities: longer navigation-duration may facilitate spatial integration processes (Thorndyke & Hayes-Roth, 1982), but may also introduce temporal distortion-related biases in distance estimation (Brunec et al., 2017, 2020; Riemer et al., 2018). Accordingly, navigation-duration is expected to affect route distance judgments and potentially influence configurational spatial representations.

#### H2

Landmark availability is expected to moderate the influence of navigation-duration on spatial knowledge processing (Wunderlich & Gramann, 2021). In metric spatial tasks, landmarks are expected to reduce time-related estimation biases and improve the accuracy of route distance judgments (Wunderlich & Gramann, 2021), particularly under short navigation-duration conditions. Landmark availability is also expected to facilitate the integration of spatial structure in sketch mapping tasks (Peer et al., 2021; Wunderlich & Gramann, 2021), whereas landmark sequence memory is expected to show relatively limited sensitivity to manipulations of navigation-duration and landmark availability.

#### H3

The joint effects of navigation-duration and landmark availability on spatial estimation will be moderated by navigational perspective, reflecting differences in the accessibility of global spatial structure and the resulting weighting of temporal versus spatial information (Bicanski & Burgess, 2020; Török et al., 2014).

#### H3a

Under an aerial perspective, where global spatial structure is readily available (Bicanski & Burgess, 2020), landmark availability—particularly in conjunction with longer navigation-duration—will yield the highest spatial accuracy, reflecting efficient integration of temporal and spatial cues (Iachini et al., 2023).

#### H3b

Under a ground-level perspective, where spatial information is locally constrained, the contribution of landmarks will be reduced, and spatial estimation will rely more strongly on temporal information, leading to a stronger effect of navigation-duration (Jovanovic & Mamassian, 2020; Török et al., 2014).

This study contributes to the understanding of cognitive map formation by examining how spatial distortions vary as a function of navigation-duration, landmark availability, and navigational perspective. The findings further suggest that spatial estimation is shaped by context-dependent interactions between temporal experience and environmental structure, rather than by spatial encoding processes alone. Practically, these results may have implications for the design of navigation and spatial learning systems that consider users’ temporal experience, perspective, and available environmental cues.

## Method

### Participants

Prior studies have shown that individuals with a poor sense of direction typically struggle to form cognitive maps (Ishikawa & Zhou, 2020). In a pilot study, we further found that individuals with relatively low spatial ability were often unable to complete the sketch mapping task. To minimize the influence of extreme scores on the experimental data and to ensure that participants were able to complete the basic spatial learning tasks, we assessed participants’ spatial ability using the Chinese adapted version of the Santa Barbara Sense of Direction Scale (SBSOD; Hegarty et al., 2002; Zhao, 2021). According to the cross cultural scoring criteria established by Montello and Xiao (2011), participants with low directional ability typically achieve scores of *M* ± SD = 2.90 ± 0.70. The present study excluded only participants with extremely poor directional ability, defined as those with an average item score below 3 and a total score below 36. Given that the present study employed a three-factor mixed experimental design, an a priori power analysis was conducted using MorePower 6.0.4 (Campbell & Thompson, 2012). Following Cohen’s (1988) conventional criterion for factorial ANOVA designs, a medium effect size (*f* = 0.25) was assumed. The analysis indicated that a minimum sample size of 128 participants would be sufficient to achieve the desired statistical power (*α* = 0.05, 1 − *β* = 0.80)*.* A total of 218 participants completed the SBSOD, of whom 154 had sufficiently good directional ability to meet the inclusion criteria. Of these, five withdrew due to perceived task difficulty, resulting in a final sample of 149 participants (91 females, 58 males; *M* ± SD = 20.18 ± 2.11). The study was approved by the Ethics Committee of Shaanxi Normal University, and all participants provided written informed consent prior to participation.

### Materials and measures

The present study employed a 2 (navigation-duration: 50-s vs. 100-s) × 2 (perspective: ground-level vs. aerial) × 2 (landmark availability: absent vs. present) mixed factorial design. In the present study, the spatiotemporal properties of navigation were operationalized through navigation-duration while route length was held constant. Because subjective time estimation is highly susceptible to preceding temporal context and adaptation effects (Ayhan et al., 2009), navigation-duration was treated as a between-subjects variable to minimize potential carryover effects on temporal processing during virtual navigation. Participants were randomly assigned to either the 50-s navigation-duration condition (*N* = 74) or the 100-s navigation-duration condition (*N* = 75). Perspective and landmark availability were treated as within-subjects variables to reduce the influence of individual differences on task performance. The dependent variables consisted of spatial judgments derived from cognitive maps, including route distance estimation, landmark sequence memory, and sketch-map accuracy reflecting overall environmental structure.

#### Stimuli

**Fig. 1:**
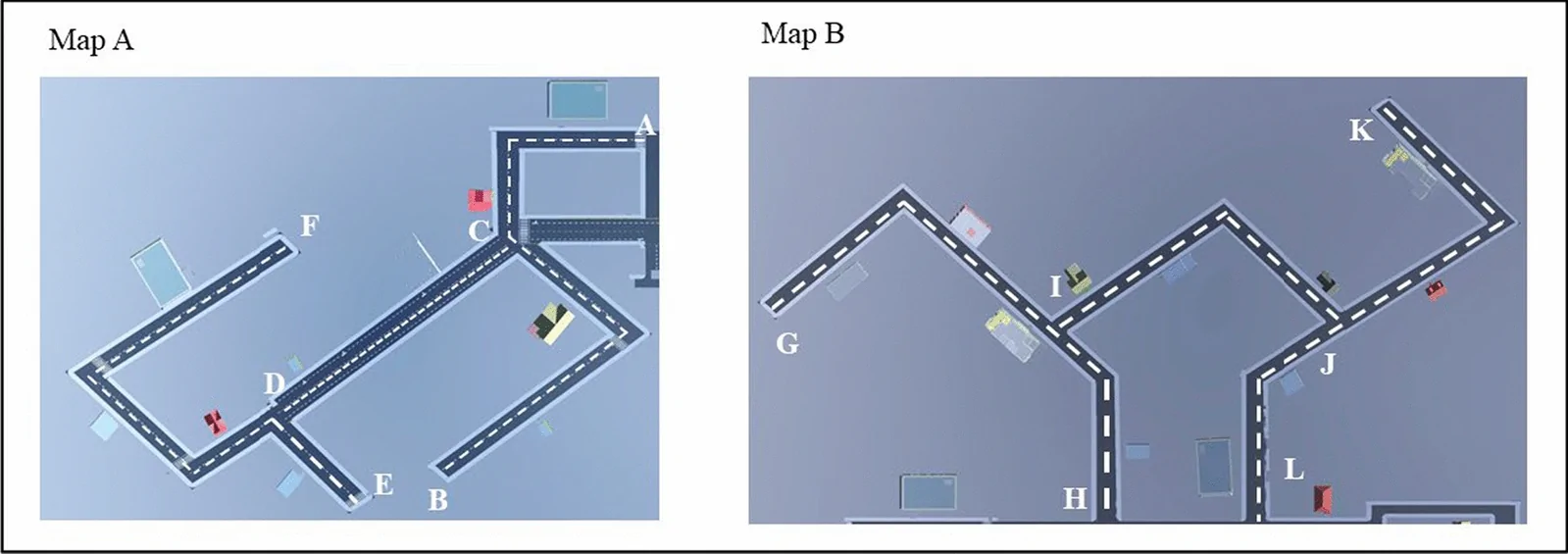
Virtual maps A and B. In map A, AB and EF are the main routes, and CD is the connecting route; in map B, GH and IJ are the main routes, and KL is the connecting route

Two virtual maps were designed to assess the participants’ acquisition of spatial knowledge. To ensure comparable memory demands, the maps were matched in terms of the number and route length of routes. Map A was presented from a ground-level perspective and consisted of three routes: AB, CD, and EF. Routes AB and EF served as the main routes, each comprising three linear segments connected by two turning points. Route CD functioned as a connecting route between the two main routes and consisted of two segments with one turning point. By contrast, Map B was presented from an aerial perspective and comprised routes GH, IJ, and KL, arranged according to the same structural logic as Map A. Overhead views of both maps are presented in Fig. 1. The virtual maps were constructed using the POLYGON City Pack in Unity 2019.4.40. For Map A (ground-level view), the camera angle was set to 60°. Navigation-duration was manipulated by varying movement speed, while maintaining constant route length. In the 100-s condition, the walking speed was set at 6 km/h; in the 50-s condition, it was set at 12 km/h. For Map B (aerial view), the camera’s field of view was set to 60°, and the viewport rectangle was set to *W* = 1, *H* = 1; the timing and speed configurations were the same as for Map A.

The landmark sequence task was implemented on the TC Lab online platform. Experiments were conducted on Apple MacBook Air (M1, CPU 2.4 GHz) computers connected to 21-inch Dell monitors (1920 × 1080 resolution, 75 Hz refresh rate). All participants completed the experiment in a quiet laboratory setting with consistent lighting conditions.

#### Measures

*Sense of direction scale* This study used the Chinese version of the Santa Barbara Sense of Direction Scale (SBSOD), originally developed by Hegarty et al. (2002) and culturally adapted by Zhao (2021). The SBSOD comprises 12 items, six of which are reverse-coded. The SBSOD has demonstrated good reliability and validity, with a Cronbach’s *α* of 0.906 in the current study.

*Perspective-taking ability scale* The perspective-taking ability scale (PTSOT) developed by Hegarty and Waller (2004) was used in this study. In this task, participants were asked to imagine themselves standing at one landmark and facing a second one, and then indicate the direction of a third target object using a circular response diagram. The test comprises eight items, each requiring a rotation of at least 90°, with correct responses evenly distributed across the four quadrants. The original scoring method calculated average angular error, with lower scores indicating higher perspective-taking ability. For ease of interpretation, the scoring was reversed for the current study: the final score was calculated as 180° minus the average angular error.

#### Tasks

Three types of spatial tasks were employed to assess different forms of spatial knowledge. The route distance estimation task measured participants’ judgments of route length, the landmark sequence task assessed memory for the sequential order of landmarks, and the sketch-mapping task evaluated the overall structural organization of the cognitive map. Detailed descriptions of the tasks are provided below.

**Fig. 2:**
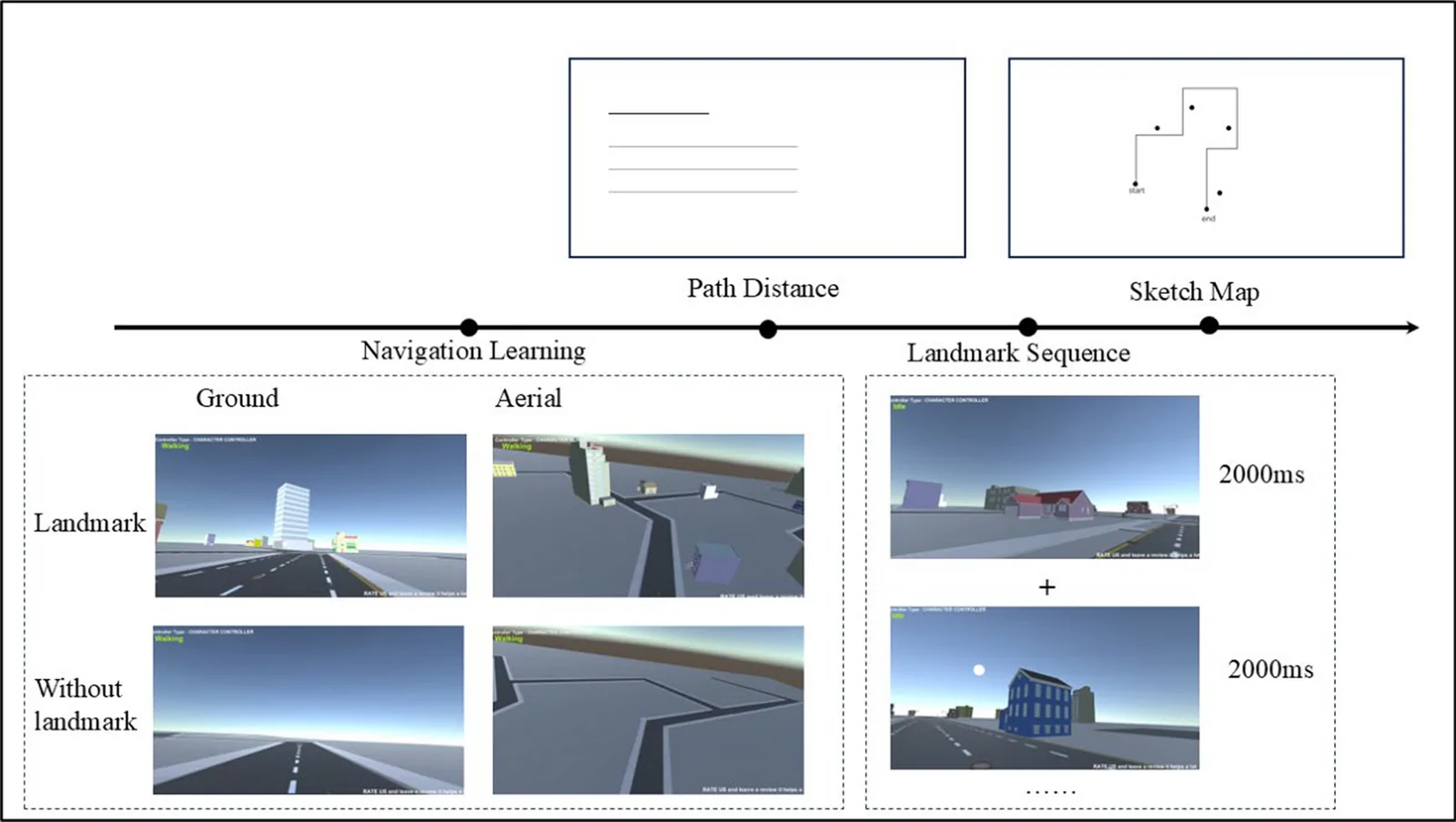
Diagram of the experimental procedure. After completing the navigation learning phase, participants sequentially performed three tasks, in a fixed order: the path distance estimation task, the landmark sequence task, and the sketch-map task

*Route distance estimation* Following the procedure of Ishikawa and Zhou (2020), participants were asked to estimate the distances between turns on each of four paths and compare them to a reference distance presented at the start, corresponding to the first traversed segment. An A4 sheet was pre-printed with a standard reference line; participants marked their estimations on parallel lines below (see Fig. 2).

*Landmark sequence task* This task, adapted from the n-back paradigm, was only administered in the landmark-present condition. Participants were presented with a randomized series of landmarks from the navigated path and asked to judge whether the currently presented landmark appeared before or after the previously presented one along the navigated path. They pressed the *Q* key to indicate *before* and the *P* key to indicate *after *(see Fig. 2). It should be noted that previous research has suggested that even in tasks requiring explicit sequential judgments, participants may spontaneously adopt survey-based, allocentric strategies rather than relying exclusively on route-based sequential encoding (Bonavita et al., 2022). Accordingly, performance on this task may reflect both sequential memory and global spatial representations.

*Sketch mapping* Participants were asked to draw the route they had learned on a blank A4 sheet, indicating the route’s shape, its start and end points, landmarks (in the landmark-present condition), and turning points. Sketch accuracy was scored independently by two doctoral students using the criteria taken from Yang (2020) (see Fig. 2).

### Procedure

Participants were randomly assigned to either the 50-s or 100-s navigation-duration condition. Before the formal experiment, participants were informed that they would complete route learning tasks across four virtual environments: ground-level perspective with landmarks, ground-level perspective without landmarks, aerial perspective with landmarks, and aerial perspective without landmarks. The presentation order of the four environments was counterbalanced across participants. The experimenter explained the procedure and administered a practice session identical to the formal task. Participants proceeded to the formal experiment only after demonstrating task comprehension and achieving at least 80% accuracy on the landmark sequence task.

During the formal experiment, participants learned each virtual environment through passive observation of navigation videos. Each navigation video was presented only once and could not be replayed. Participants were instructed to memorize the route structure as accurately as possible and, in the landmark-present condition, to additionally remember the positional relations among landmarks. After learning each environment, participants sequentially completed the route distance estimation task, landmark sequence task, and sketch mapping. During the sketch mapping, participants continuously extended the same map across route learning phases. Specifically, each newly learned route had to be drawn beginning from the endpoint of the previously drawn route, such that participants gradually constructed a single integrated map containing two main routes and one connecting route rather than separate route sketches. After completing all navigation learning and spatial tasks, participants completed the Sense of Direction Scale and the perspective-taking ability scale.

### Data analysis

All reported data are available via osf.io(https://osf.io/v8m2g/overview?view_only=7042af7096f94eca959388885958f1de). The alpha level was set at 0.05. For repeated-measures and factorial ANOVAs, effect sizes are reported as partial eta squared (*η*_p_^2^), along with 95% confidence intervals (CIs). For correlation analyses, Pearson’s *r* values with 95% CIs are reported.

For the route distance estimation task, analyses focused on judgment error in estimated path distances. Path distance judgment error was quantified as the deviation between participants’ estimated relative path lengths and the corresponding actual relative path lengths. For each path segment, the ratio between the participants’ estimated length and a standard reference path was compared with the ratio between the actual path length and the same reference. Following Ishikawa and Zhou (2020), judgment error for each segment (${d}_{i}$) was computed as:

$${{d}}_{i}=\frac{{{L}_{j}}_{i}}{{L}_{s}}-\frac{{{L}_{o}}_{i}}{{L}_{s}}$$

$${D}_{1}=\frac{1}{n}{\sum}_{i=1}^{n}{d}_{i}$$

where ${L}_{j}$ denotes the estimated path length, ${L}_{o}$ denotes the actual path length, ${L}_{s}$ denotes the standard reference path, and ${D}_{1}$ represents the mean judgment bias across all segments. Positive values indicate overestimation of relative path length, whereas negative values indicate underestimation. Deviation scores (${D}_{1}$) were subjected to repeated-measures ANOVA to assess differences in route knowledge. Error values closer to zero (i.e., smaller absolute error) indicate higher judgment accuracy. A three-factor mixed-design ANOVA was conducted on route distance judgment error, with navigation-duration as the between-subjects factor and landmark availability and perspective as within-subjects factors.

For the landmark sequence task, accuracy was calculated as the proportion of correct responses relative to the total number of trials. Because this task was administered only under landmark-present conditions, a two-factor mixed-design ANOVA was conducted, with navigation-duration as the between-subjects factor and perspective as the within-subjects factor.

For the sketch-mapping task, sketch accuracy was calculated based on the scoring rubric developed by Yang. Scores ranged from 0 to 36 (landmark-absent condition) and 0 to 46 (landmarks present condition). Accuracy was defined as the ratio between the participant’s obtained score and the maximum possible score, with values closer to 1 indicating greater accuracy. Sketch-mapping accuracy was analyzed using a three-factor mixed-design ANOVA, with navigation-duration as the between-subjects factor and landmark availability and perspective as within-subjects factors. Prior to inferential analyses, assumptions of normality and homogeneity of variance were assessed using Kolmogorov–Smirnov tests, Shapiro–Wilk tests, visual inspection of Q–Q plots, skewness/kurtosis indices, and Levene’s tests. Because ANCOVA analyses included SBSOD and PTSOT scores as covariates, only participants with complete questionnaire data were included in these analyses. To evaluate whether questionnaire missingness was systematically associated with experimental group assignment, a Pearson’ s Chi-square test of independence was conducted to compare  questionnaire completion status (complete vs. missing) across navigation-duration conditions. Additional mixed-design analyses of covariance (ANCOVAs) were conducted using SBSOD and PTSOT scores as covariates.

Correlations among the scores for the three spatial tasks (path distance estimation, landmark sequence accuracy, and sketch accuracy) were calculated and regression analyses were conducted to assess how route and landmark knowledge predicted global knowledge. We further investigated whether individual spatial ability predicted task performance. Data were analyzed using IBM SPSS 27.0.

## Results

### Path distance estimation

Prior to conducting the mixed-design ANOVAs, normality was assessed using the Kolmogorov–Smirnov and Shapiro–Wilk tests, together with visual inspection of Q–Q plots and boxplots. Although several conditions showed significant Shapiro–Wilk statistics, visual inspection suggested only minor deviations from normality. Levene’s tests indicated that the assumption of homogeneity of variance was satisfied across all conditions (*p* > 0.05). Given the relatively large and balanced sample sizes, mixed-design ANOVA was considered robust to these minor violations.

Descriptive statistics for all conditions are presented in Table 1. To ensure equivalence between the navigation-duration groups in terms of spatial cognitive ability, independent-samples *t*-tests were conducted using navigation-duration as the grouping variable and scores on the Sense of Direction Scale and the perspective-taking ability scale as dependent variables. The results indicated that neither perspective-taking ability, *t*(115) = − 0.43, *p* = 0.668, nor sense of direction, *t*(147) = − 0.392, *p* = 0.696, differed significantly between the two navigation-duration groups.

**Table 1:** Descriptive statistics across all levels of navigation-duration, perspective, and landmark condition

Variables	Perspective	Landmarks	*M*	*SD*
*Path distance task*				
50-s	Ground	Absent	− 0.002	0.263
		Present	− 0.001	0.168
	Aerial	Absent	− 0.085	0.217
		Present	− 0.161	0.208
100-s	Ground	Absent	− 0.100	0.246
		Present	0.002	0.179
	Aerial	Absent	− 0.069	0.182
		Present	− 0.091	0.175
*Landmark sequence task*				
50-s	Ground		0.475	0.179
	Aerial		0.457	0.248
100-s	Ground		0.4376	0.133
	Aerial		0.4372	0.222
*Sketch-map task*				
50-s	Ground	Absent	0.711	0.152
		Present	0.733	0.126
	Aerial	Absent	0.674	0.168
		Present	0.763	0.156
100-s	Ground	Absent	0.789	0.154
		Present	0.779	0.144
	Aerial	Absent	0.767	0.166
		Present	0.832	0.152

**Fig. 3:**
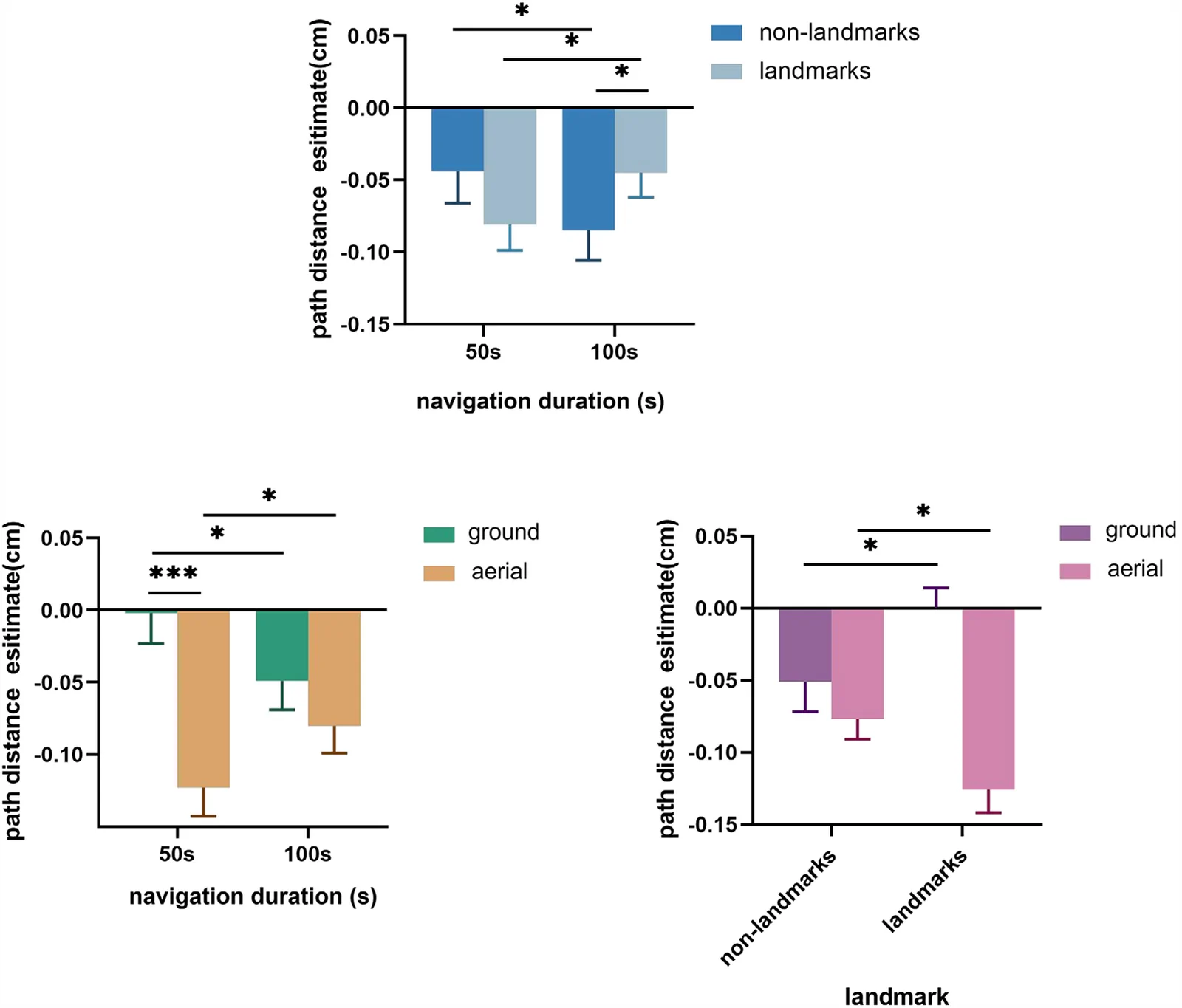
Interaction plots for each experiment. **A** Interaction between navigation-duration and landmark condition; **B** interaction between navigation-duration and perspective; **C** interaction between perspective and landmark condition

A three-way mixed-design ANOVA was conducted on path distance judgment error, with navigation-duration (50-s vs. 100-s) as the between-subjects factor and landmark condition and perspective as within-subjects factors. Results revealed no significant main effects of landmark availability, *F*(1, 147) = 0.008, *p* = 0.930; nor of navigation-duration, *F*(1, 147) = 0.009, *p* = 0.926. However, a significant main effect of perspective was found, *F*(1, 147) = 27.148, *p* < 0.001, *η*_p_^2^ = 0.156, 95%CI = [0.063, 0.262], indicating that participants produced path distance judgment errors closer to zero under the ground-level perspective (*M* =− 0.025, SD = 0.015) than under the aerial view (*M* =− 0.101, SD = 0.014). A significant interaction between navigation-duration and landmark condition was observed, *F*(1, 147) = 8.035, *p* = 0.005, *η*_p_^2^ = 0.052, 95%CI = [0.007, 0.106]. Simple-effects analyses showed that under the 100 s navigation-duration condition, judgment error was smaller in the landmark-present condition (*M* = − 0.045, SD = 0.017) than in the landmark-absent condition (*M* = − 0.085, SD = 0.021), *F*(1, 147) = 4.481, *p* = 0.036, *η*_p_^2^ = 0.030. No significant landmark effect was observed under the 50-s condition. (*M*_present_ = − 0.081, SD_present_ = 0.018, *M*_absent_ = − 0.044, SD_absent_ = 0.022). There was also a significant interaction found between navigation-duration and perspective, *F*(1, 147) = 9.548, *p* = 0.002, *η*_p_^2^ = 0.061, 95%CI = [0.012, 0.118]. Under the 50-s condition, judgment error was smaller under the ground-level perspective (*M* = − 0.002, SD = 0.021) than under the aerial perspective (*M* = − 0.123, SD = 0.020), *F*(1, 147) = 32.902, *p* < 0.001, *η*_p_^2^ = 0.183. No significant perspective effect emerged between the ground-level perspective (*M* = − 0.049, SD = 0.020) and the aerial perspective (*M* =− 0.080, SD = 0.019), *F*(1, 147) = 2.359, *p* = 0.127 under the 100-s condition. A significant interaction was also observed between landmark availability and perspective, *F*(1, 147) = 17.123, *p* < 0.001, *η*_p_^2^ = 0.104, 95%CI = [0.042, 0.193]. Post-hoc tests showed that under the ground-level condition, path distance judgment error was significantly smaller in the landmark-present condition (*M* = 0.000, SD = 0.014) than in the landmark-absent condition (*M* = − 0.051, SD = 0.021), *F*(1, 147) = 6.495, *p* = 0.012, *η*_p_^2^ = 0.042, 95%CI = [0.005, 0.093]. Conversely, under the aerial view condition, path distance judgment error was significantly smaller in the landmark-absent condition (*M* = − 0.077, *SD* = 0.016) than in the landmark-present condition (*M* = − 0.126, SD = 0.016), *F*(1, 147) = 9.284, *p* = 0.003, *η*_p_^2^ = 0.059, 95%CI = [0.011, 0.116]. The three-way interaction among perspective, landmark presence, and navigation-duration did not have a significant effect, *F*(1, 147) = 1.016, *p* = 0.315. Interaction plots are presented in Fig. 3.

### Landmark sequence task

Assumption testing for landmark sequence accuracy indicated mild deviations from normality in several conditions; however, skewness and kurtosis values remained within acceptable ranges. Levene’s test indicated a minor violation of homogeneity for the ground-view perspective condition, *F*(1, 147) = 4.880, *p* = 0.029, whereas the aerial view perspective condition satisfied the assumption, *F*(1, 147) = 0.997, *p* = 0.320. Descriptive statistics are presented in Table 1. The mixed-design ANOVA was therefore considered robust to these minor violations. A two-way mixed-design ANOVA was conducted with navigation-duration (50-s vs. 100-s) as between-subjects factor and perspective (ground vs. aerial) as within-subjects factor. Neither the main effect of perspective, *F*(1, 147) = 0.159, *p* = 0.691, nor the main effect of navigation-duration, *F*(1, 147) = 1.452, *p* = 0.230, reached significance. The interaction between navigation-duration and perspective was also non-significant, *F*(1, 147) = 0.181, *p* = 0.671. A linear mixed-effects model including participants as a random effect was additionally conducted. The model revealed no significant effects of perspective (*β* = − 0.038, *p* = 0.719), navigation-duration (*β* = − 0.001, *p* = 0.270), or their interaction (*β* = 0.000, *p* = 0.844). These findings were consistent with the ANOVA results.

### Sketch mapping

Assumption testing indicated no severe deviations from normality. Although several Shapiro–Wilk tests reached significance, skewness and kurtosis values remained within acceptable ranges across conditions (|skewness| ≤ 1.07, |kurtosis| ≤ 3.16). Levene’s tests further indicated that the assumption of homogeneity of variance was satisfied across all conditions (all *p* > 0.05). Therefore, the mixed-design ANOVA was considered appropriate for the present analyses. Descriptive statistics are presented in Table 1. A three-way mixed-design ANOVA was conducted on participants’ accuracy on the sketch-map task, with navigation-duration (50-s vs. 100-s) as the between-subjects factor, and perspective (ground vs. aerial) and landmark condition (landmarks present vs. absent) as within-subjects factors. The main effect of perspective was not significant, *F*(1, 147) = 0.255, *p* = 0.615. However, the main effect of landmark availability was significant, with sketch mapping accuracy being significantly higher in the landmark-present condition (*M* = 0.777, SD = 0.010) than in the landmark-absent condition (*M* = 0.735, SD = 0.010), *F*(1, 147) = 19.159, *p* < 0.001, *η*_p_^2^ = 0.115, 95%CI = [0.061, 0.183]. The main effect of navigation-duration was also significant, with participants showing higher sketch-map accuracy in the 100-s navigation-duration condition (*M* = 0.792, SD = 0.012) than in the 50-s condition (*M* = 0.720, SD = 0.013), *F*(1, 147) = 16.756, *p* < 0.001, *η*_p_^2^ = 0.102, 95%CI = [0.051, 0.169].

**Fig. 4:**
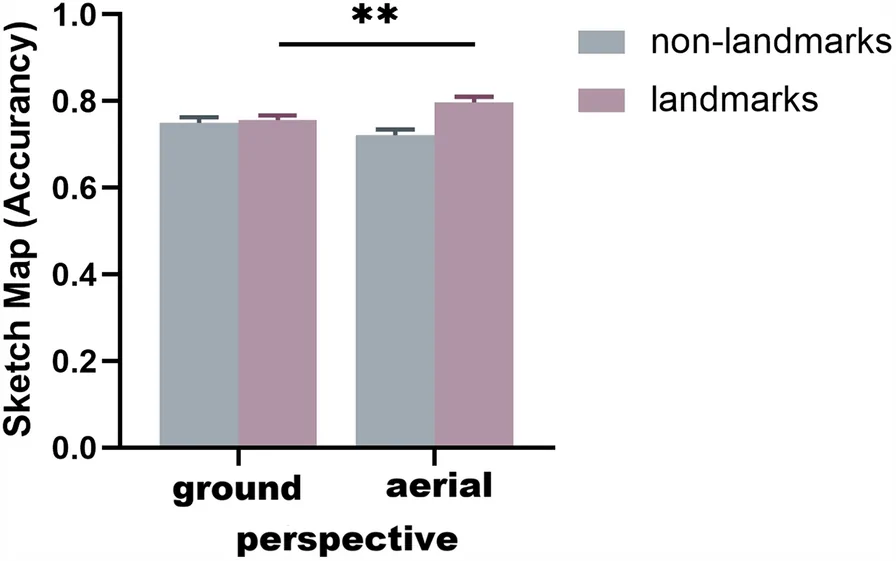
Interaction between perspective and landmark condition

Neither the interaction between perspective and navigation-duration, *F*(1, 147) = 0.606, *p* = 0.438, nor the interaction between landmark condition and navigation-duration, *F*(1, 147) = 2.152, *p* = 0.144, reached significance. However, a significant interaction between perspective and landmark condition emerged, *F*(1, 147) = 15.537, *p* < 0.001, *η*_p_^2^ = 0.096, 95%CI = [0.046, 0.163]. Post-hoc comparisons revealed that under the landmark-absent condition, sketch-map accuracy did not differ significantly between the ground-level perspective (*M* = 0.750, SD = 0.013) and the aerial perspective (*M* = 0.721, SD = 0.014), *F* (1, 147) = 3.109, *p* = 0.080. Under the landmark-present condition, sketch-map accuracy under the ground-level perspective (*M* = 0.756, SD = 0.011) was significantly lower than under the aerial perspective (*M* = 0.797, SD = 0.013), *F*(1, 147) = 8.773, *p* = 0.004, *η*_p_^2^ = 0.056, 95%CI = [0.010, 0.111]. The effect of the three-way interaction between perspective, landmark condition, and navigation-duration was not significant, *F*(1, 147) = 0.073, *p* = 0.788.The interaction plot is shown in Fig. 4.

Because ANCOVA analyses required complete SBSOD and PTSOT data, only participants with available questionnaire data were included (*N* = 111). A Pearson’s chi-square test indicated that questionnaire completion was not associated with navigation-duration condition, *χ*^2^(1, 149) = 0.19, *p* = 0.66, Cramér’s *V* = 0.04, suggesting that missing questionnaire data did not systematically bias experimental group assignment. Additional mixed-design ANCOVAs were conducted using SBSOD and PTSOT scores as covariates. No significant interactions involving SBSOD were observed (all *p* > 0.05), indicating that self-reported sense of direction did not differentially influence sketch-map accuracy across experimental conditions. For the PTSOT, the interaction between perspective and perspective-taking ability approached significance, *F*(1, 109) = 3.284, *p* = 0.073, *η*^2^ = 0.029, suggesting that perspective-taking ability may partially moderate the effect of navigational perspective on cognitive map construction. All remaining interactions involving PTSOT were non-significant (all *p* > 0.05). Overall, including individual-difference covariates did not substantially alter the primary pattern of results.

### Relationships among route knowledge, landmark knowledge, and survey knowledge

Separate Pearson correlation analyses were conducted to examine the relationships among route knowledge, landmark knowledge, and survey knowledge under the ground-level and aerial perspective conditions.

**Fig. 5:**
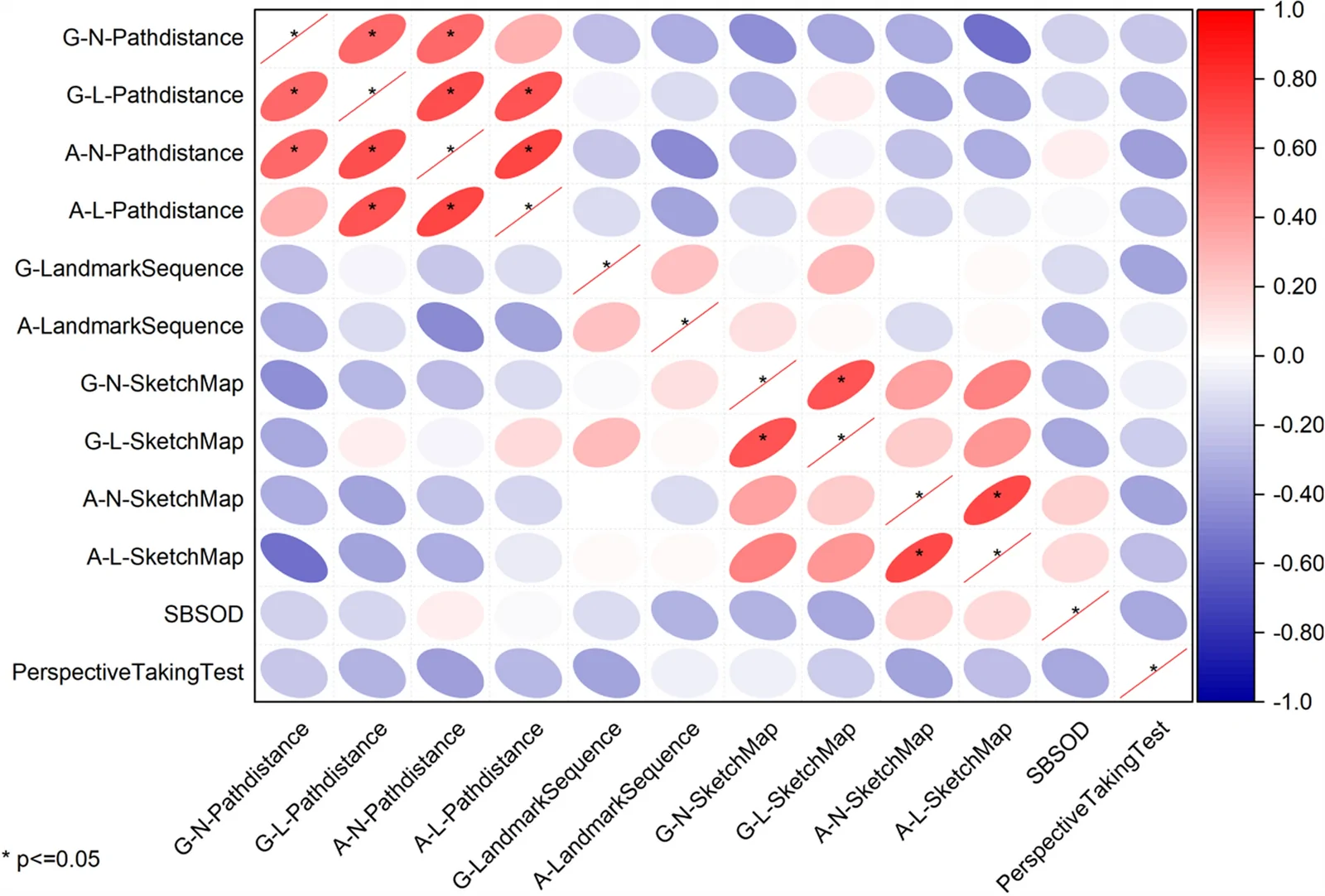
Correlations among all variables. *Note* G-N = ground perspective–no-landmarks, G-L = ground perspective–landmarks present, A-N = aerial perspective–no-landmarks, A-L = aerial perspective–landmarks present. *p ≤ 0.05

Under ground-level perspective, sketch-map accuracy in the landmark-absent condition was not significantly correlated with path distance judgment error in either the landmark-absent condition (*r*(146) =− 0.099, *p* = 0.230) or the landmark-present condition (*r*(147) = 0.042, *p* = 0.615), nor was it correlated with landmark sequence accuracy (*r*(147) = 0.037, *p* = 0.653). In contrast, under the landmark-present condition, sketch-map accuracy showed significant positive correlations with both path distance judgment error in the landmark-present condition (*r*(148) = 0.247, *p* = 0.002, 95%CI = [0.090, 0.391]) and landmark sequence accuracy (*r*(148) = 0.249, *p* = 0.002, 95%CI = [0.093, 0.394]). No significant correlation were observed between sketch-map accuracy and path distance judgment error from the landmark-absent condition (*r*(146) = − 0.070, *p* = 0.401). Together, these findings suggest that under the ground-level perspective, survey knowledge was associated with both route-based metric knowledge and landmark sequence knowledge only when landmarks were available. In the absence of landmarks, sketch-map performance appeared to be relatively independent of other spatial knowledge components. Under the aerial perspective, sketch-map accuracy in the landmark-absent condition was not significantly correlated with path distance judgment error in either the landmark-absent condition (*r*(147) = 0.024, *p* = 0.772) or the landmark-present condition (*r*(147) = 0.085, *p* = 0.303), nor with landmark sequence accuracy (*r*(148) = − 0.005, *p* = 0.949). However, in the landmark-present condition, sketch-map accuracy was significantly correlated with path distance judgment error (*r*(147) = 0.179, *p* = 0.029, 95%CI = [0.018, 0.330]), whereas its correlation with landmark sequence accuracy (*r*(148) = 0.108, *p* = 0.190) and path distance judgment error in the landmark-present condition did not reach statistical significance (*r*(148) = − 0.005, *p* = 0.950). This pattern suggests that, under aerial perspective conditions, route-based metric knowledge—but not landmark sequence knowledge—is preferentially integrated into survey-level representations when landmark information is present. With respect to individual differences, SBSOD scores were significantly correlated with sketch-map accuracy under the aerial perspective, both in the landmark-absent condition (*r*(147) = 0.254, *p* = 0.002, 95%CI = [0.097, 0.398]) and in the landmark-present condition (*r*(147) = 0.176, *p* = 0.032, 95%CI = [0.016, 0.328]). In contrast, PTSOT scores were not significantly correlated with sketch-map accuracy or path distance judgment error in either perspective condition. A full correlation matrix for all variables is presented as a heatmap in Fig. 5.

**Fig. 6:**
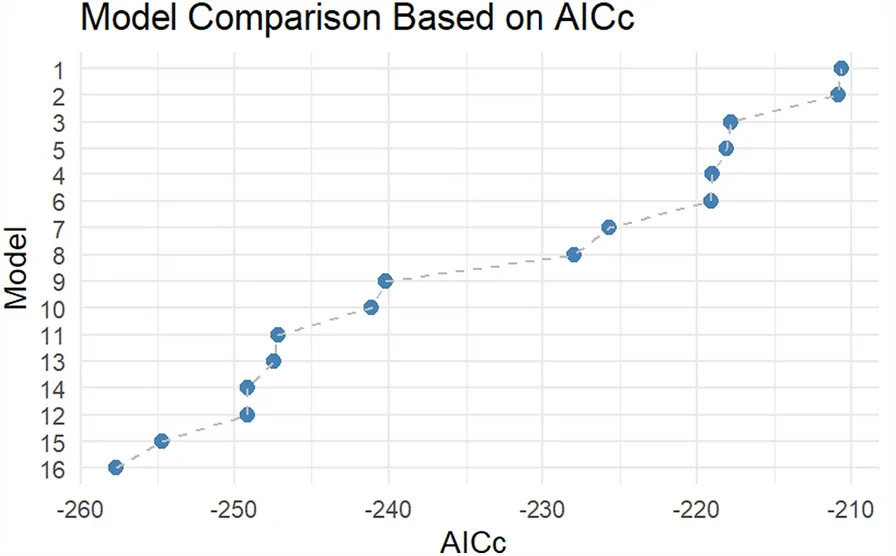
Model comparison based on AICc

**Fig. 7:**
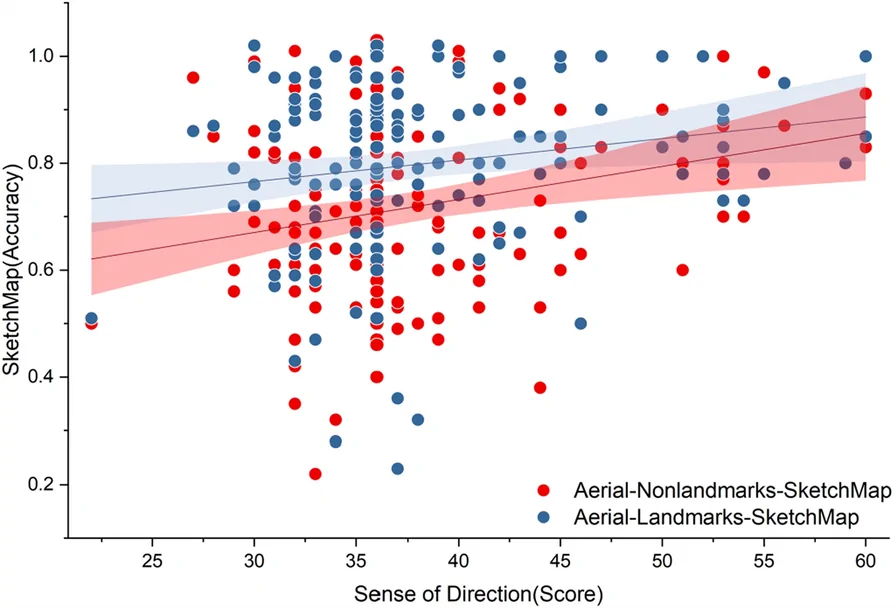
Scatter plot for sense of direction scores and sketch-map accuracy

 A linear mixed-effects model was constructed to examine predictors of sketch-map accuracy, including Path Distance, Landmark Sequence, Sense of Direction, Perspective, and navigation-duration as fixed effects, with participants included as a random intercept. Model selection was based on AICc comparisons among candidate models. The AICc-based selection yielded multiple candidate models, whose ranked AICc values are presented in Fig. 6. The model with the lowest AICc value included four fixed effects (Landmark Sequence, Sense of Direction, navigation-duration, and Perspective) and a random intercept for participants. This model yielded a marginal *R*^2^ of 0.157, indicating that the fixed effects accounted for approximately 15.7% of the variance, and a conditional *R*^2^ of 0.481, suggesting that the model explained 48.1% of the variance when both fixed and random effects were taken into account. Notably, although path distance judgment error showed condition-dependent correlations with sketch-map accuracy, it did not explain additional unique variance once landmark sequence knowledge, perspective, and navigation-duration were simultaneously taken into account. Detailed regression coefficients are given in Table 2, and scatterplots illustrating the relationships among the predictors are presented in Fig. 7.

**Table 2:** Summary of fixed effects in the final linear mixed-effects model predicting sketch-mapping performance

Predictor	*β*	SE	d*f*	*t*	*p*
(Intercept)	0.427	0.066	180.9	6.443	< 0.001^***^
Landmark sequence	0.101	0.045	264.6	2.269	0.024^*^
Sense of direction	0.005	0.002	145.2	3.334	0.001^**^
navigation-duration	0.073	0.022	144.8	3.335	0.001^**^
Perspective	0.087	0.015	143.8	5.989	< 0.001^***^

*R*^*2*^*m* = 0.157, *R*^*2*^*c* = 0.481

*p < 0.05, **p < 0.01, ***p < 0.001

## Discussion

The present study examined how navigation-duration, landmark availability, and navigational perspective jointly influence different forms of spatial knowledge during cognitive map formation. The results demonstrated that the effects of these factors on spatial knowledge processing were both task-specific and context-dependent. Overall, navigation-duration primarily influenced metric spatial processing, particularly route distance estimation, whereas its effects on landmark sequence memory and configurational spatial processing in sketch mapping were relatively limited. Landmark availability and navigational perspective further moderated the influence of navigation-duration on spatial judgments; however, these effects were mainly reflected in multiple two-way interactions rather than a unified three-way integrative mechanism. Specifically, landmarks altered the direction of the effect of navigation-duration on distance estimation, whereas the interactive roles of landmarks and navigation-duration differed across metric judgment tasks and structural integration tasks under different perspective conditions. The following sections discuss the distinct roles of navigation-duration, landmarks, and navigational perspective in cognitive map formation.

### The influence of the spatiotemporal properties of navigation on spatial knowledge in cognitive maps

The present study examined the role of the spatiotemporal properties of navigation across different forms of spatial processing. The findings revealed a differential pattern across tasks: navigation-duration exerted a stronger and more consistent influence on path distance estimation than on landmark sequence memory and sketch-map performance. However, sketch-map accuracy was also influenced by navigation-duration—with higher accuracy following longer navigation—indicating that structural representations were not entirely independent of temporal manipulation. Specifically, in the route distance estimation task, navigation-duration produced a robust bias effect on the processing of metric information. Participants in the shorter navigation-duration condition (50-s) generated distance estimates that were closer to the objective spatial layout, whereas participants in the longer navigation-duration condition (100-s) exhibited larger judgment errors. Although the effect of navigation-duration was moderated by both perspective and landmark availability, the overall pattern remained relatively stable across conditions. In the sketch-mapping task, longer navigation-duration was associated with higher sketch accuracy, whereas no significant main effect of navigation-duration emerged in the landmark sequence task.

These findings are consistent with one of the two possibilities outlined in H1. Our hypothesis acknowledged that longer navigation-duration could either facilitate spatial integration or introduce temporal distortion-related biases into distance estimation. The present results align with the latter possibility: longer navigation-duration increased distance estimation error rather than improving accuracy. This pattern suggests that, under the current experimental conditions, prolonged navigation may have increased susceptibility to temporal distortion during spatial judgment, rather than providing additional opportunities for spatial integration. Previous research has shown that subjective temporal experience during navigation does not always correspond to objective duration, and that extended movement may induce duration-related biases (Arnold et al., 2016; Burgess et al., 2001). Under such conditions, spatial distance judgments may become increasingly influenced by subjective temporal processing, thereby producing systematic metric errors. From this perspective, the larger distance estimation errors observed under longer navigation-duration may reflect biases associated with sustained temporal processing during navigation. Importantly, however, the present study did not directly measure subjective time perception. Therefore, temporal distortion should be regarded only as a theoretically plausible interpretation rather than a directly verified mechanism. By contrast, navigation-duration exerted comparatively limited effects on tasks involving structural spatial representations. Previous research suggests that the organization of spatial layouts depends more heavily on visual input, path integration, and landmark-based reference information (Fischler-Ruiz et al., 2021; Kozhevnikov & Puri, 2023). Accordingly, although navigation-duration altered metric judgments of spatial distance, it did not substantially disrupt the overall structural organization of spatial representations. This interpretation is broadly consistent with the null effects observed in the landmark sequence task and the relatively limited effects observed in sketch mapping. Together, these findings suggest that navigation-duration influenced both metric and structural aspects of spatial representation, albeit to different degrees. Metric distance judgments were more strongly and systematically affected by temporal variation, whereas configurational representations showed a more moderate, yet still observable, sensitivity to navigation-duration. This pattern points toward differential rather than independent effects of temporal information across forms of spatial knowledge.

### Landmarks and the spatiotemporal properties of navigation in cognitive map formation

As discussed in “The influence of the spatiotemporal properties of navigation on spatial knowledge in cognitive maps” section, the present study found that navigation-duration primarily influenced metric spatial judgments, with longer navigation-duration increasing distance estimation error. Importantly, however, this temporal effect did not remain stable across all environmental conditions. Further analyses demonstrated that the presence of landmarks significantly altered the manner in which navigation-duration influenced path distance judgments, indicating that the effect of navigation-duration on spatial judgment was moderated by environmental structure. Specifically, the interaction between landmark availability and navigation-duration observed in the route distance estimation task revealed that the typical time-distance coupling pattern—namely, that longer navigation-duration induces larger distance estimation bias under landmark-absent conditions—was attenuated or even reversed when landmarks were available.Under the landmark-present condition, participants in the 100-s condition produced distance estimates that were closer to the actual spatial layout, whereas larger estimation errors emerged under the 50-s condition. Notably, landmarks not only reduced the magnitude of the navigation-duration effect but also changed its direction. Under landmark-absent conditions, longer navigation-duration was associated with greater distance estimation error, consistent with the general pattern described in “The influence of the spatiotemporal properties of navigation on spatial knowledge in cognitive maps” section. In contrast, when landmarks were present, this pattern reversed, such that longer navigation-duration resulted in more accurate distance judgments. In the sketch-mapping task, landmark availability produced a significant main effect but did not interact significantly with navigation-duration. Similarly, no significant effects involving navigation-duration were observed in the landmark sequence task.

These findings support Hypothesis 2, namely that landmark availability moderates the relationship between navigation-duration and spatial judgment. Across the three spatial tasks, the interaction between landmarks and navigation-duration was primarily observed in metric spatial processing, particularly route distance estimation, and did not generalize to sequence memory retrieval or structural integration tasks. One possible interpretation is that landmarks altered how temporal information was organized during navigation. Previous research has suggested that perceived navigation-duration is influenced not only by continuous self-motion cues such as optic flow, but also by how navigational experience is segmented into meaningful event units (Jeunehomme & D’Argembeau, 2020; Rhodes, 2018; Shin & DuBrow, 2021). According to event segmentation theory, salient environmental cues may partition continuous experience into more discrete event units, thereby influencing the encoding and retrieval of temporal information (Shin & DuBrow, 2021). When considered together with the temporal compression account discussed in “The influence of the spatiotemporal properties of navigation on spatial knowledge in cognitive maps” section, this framework may help explain how landmarks modulated distance estimation performance during extended navigation. The temporal compression account itself does not specify the conditions under which temporal distortions become stronger or weaker. Event segmentation theory provides one possible account of these boundary conditions by proposing that salient environmental nodes, such as landmarks, may reorganize continuous navigation into more structured event sequences (Jeunehomme & D’Argembeau, 2020; Rhodes, 2018; Shin & DuBrow, 2021). Event segmentation reflects an ongoing inferential process through which individuals determine the current event state, rather than merely responding to perceptual change (Shin & DuBrow, 2021). When landmarks function as stable event cues, participants may encode extended navigation as a series of segmented events rather than as a single undifferentiated temporal stream. Such restructuring may subsequently reduce the accumulation of time-related estimation bias during long-duration navigation. Importantly, however, the present study did not directly measure subjective temporal perception or event segmentation processes. Therefore, these interpretations should be regarded as theoretically plausible accounts that are broadly consistent with the observed behavioral patterns rather than as directly verified mechanisms. The facilitative effect of landmarks observed in the sketch-mapping task is likewise consistent with the broader idea that environmental structure constrains cognitive map formation, although the precise cognitive processes underlying this effect remain to be clarified in future research.

### The effects of perspective, spatiotemporal properties of navigation, and landmarks on cognitive map formation

This section further examines how perspective, landmarks, and navigation-duration jointly influenced performance across different spatial tasks, with the aim of clarifying how distinct forms of spatial knowledge are processed during cognitive map formation. Although the three-way interaction among navigation-duration, perspective, and landmark availability was not significant across tasks, multiple significant two-way interactions were observed. This pattern suggests that the effects of temporal cues, environmental landmarks, and navigational perspective on spatial cognition are condition dependent rather than governed by a fully integrated three-factor mechanism.

In the route distance estimation task, a significant interaction emerged between navigation-duration and perspective. Under the ground-level perspective, longer navigation-duration increased path distance estimation error, whereas under the aerial perspective, longer navigation-duration instead improved estimation accuracy. In addition, a significant interaction was observed between perspective and landmark availability. Under the ground-level perspective, distance estimation error was significantly smaller when landmarks were present than when landmarks were absent. In contrast, the opposite pattern emerged under the aerial perspective, where distance judgments were more accurate in the absence of landmarks. By comparison, the landmark sequence task revealed no significant effects involving navigation-duration, perspective, or landmarks, suggesting that this task was relatively insensitive to the experimental manipulations. Similarly, the sketch-mapping task did not reveal a significant three-way interaction or a main effect of perspective. However, a significant interaction between perspective and landmark availability was observed: under landmark-present conditions, sketch-map accuracy was significantly higher under the aerial perspective than under the ground-level perspective, whereas no significant perspective difference emerged under landmark-absent conditions.

Together, these findings suggest that the interaction between perspective and landmarks varied as a function of task demands. In the route distance estimation task, the aerial perspective attenuated the estimation bias associated with navigation-duration and altered the functional role of landmarks. In the sketch-mapping task, however, landmarks facilitated performance only under the aerial perspective. Importantly, the direction of these effects appeared to depend on the type of spatial knowledge required by the task—whether participants were estimating metric distance or integrating global environmental structure. One possible interpretation is that perspective altered the spatial reference-frame participants relied upon during navigation, thereby changing the weighting of temporal and environmental information during spatial judgment. However, because the present study did not directly measure reference-frame transformation, subjective temporal perception, or event segmentation, the following interpretations should be regarded as theoretically plausible accounts rather than direct demonstrations of underlying mechanisms. Under the ground-level perspective, participants may have relied more heavily on egocentric, body-centered spatial processing. Previous research has shown that spatial updating during ground-level navigation depends strongly on the integration of visual and vestibular information, with self-motion signals contributing to displacement estimation and path distance computation (Zhou & Gu, 2023; Cheng Gu, 2018). Within this framework, navigation-duration may exert a stronger influence on metric judgments. Landmarks, in turn, may help organize continuous navigational experience into more stable segments (Peer et al., 2021; Shin & DuBrow, 2021), thereby reducing time-related estimation bias during extended navigation. By contrast, the aerial perspective likely promoted a more allocentric, environment-centered mode of processing. Because aerial views directly provide global layout and topological information, spatial judgments may depend less on temporally accumulated self-motion cues and more on stable structural relations (Bicanski & Burgess, 2020; Kozhevnikov & Puri, 2023). This interpretation is consistent with the reduced influence of navigation-duration observed under the aerial perspective. It may also help explain why landmarks no longer facilitated metric judgments under aerial viewing conditions. When global structural information is already highly accessible, additional landmark cues may contribute relatively little to distance inference and may even interfere with global spatial processing by increasing competition among local visual cues. A similar framework may explain the interaction observed in the sketch-mapping task. Sketch mapping requires the integration of global spatial relations across the environment. Under the aerial perspective, participants could access configurational information more directly, facilitating the construction of allocentric representations (Thorndyke & Hayes-Roth, 1982). In this context, landmarks may have functioned as stable spatial anchors that supported the organization of global spatial structure (Wunderlich & Gramann, 2021). Consequently, the combination of aerial perspective and landmark availability produced the highest sketch-map accuracy. Without landmarks, the aerial perspective still provided global structural information, but lacked stable anchoring references that could support spatial integration. Importantly, these findings suggest that the functional role of landmarks differed across task demands. In metric judgment tasks, landmarks may have reduced temporal estimation bias by structuring navigational experience, whereas in structural integration tasks they may have served as spatial anchors supporting configurational organization. In addition, the null effects in the landmark sequence task further suggest that sequential order processing may be relatively less sensitive to these manipulations. One possible explanation comes from Bonavita et al. (2022), who reported that landmark-ordering performance was more strongly associated with survey knowledge than with route learning performance. This finding suggests that participants may spontaneously rely on allocentric, map-like representations even when performing sequential-order tasks. Applying this interpretation to the present study, participants may not have relied exclusively on sequential encoding during landmark-order judgments, potentially explaining why the task showed limited sensitivity to the experimental manipulations. Nevertheless, these interpretations remain theoretical. The present findings only demonstrate that landmarks conditionally altered the relationship between navigation-duration and spatial judgment, and that this modulation varied across task contexts. Whether these effects are genuinely mediated by event segmentation, temporal encoding, or anchoring processes remains to be tested in future studies using direct process-level measures.

Overall, the absence of a three-way interaction suggests that navigation-duration, landmarks, and perspective do not operate through a single unified mechanism. Instead, distortions during cognitive map formation appear to depend on the dynamic weighting of temporal, self-motion, and structural information under different task and reference-frame conditions.

### Route and landmark knowledge as conditional predictors of survey knowledge

The present results elucidate the conditions under which route knowledge and landmark knowledge contribute to survey knowledge. Across both correlation and regression analyses, the findings converge to show that the integration of local spatial knowledge into global representations is selective rather than automatic, and is critically constrained by perspective and landmark availability.

At the correlational level, sketch-map accuracy exhibited perspective and condition-dependent associations with route and landmark knowledge. Under the ground-level perspective, survey knowledge was associated with route- and landmark-based information only when landmarks were present, suggesting that stable environmental anchors may support global integration during egocentric navigation. Under an aerial perspective, survey performance was unrelated to route or landmark knowledge in the absence of landmarks, but selectively linked to path distance estimation when landmarks were available, suggesting a preferential integration of global metric information over sequential landmark encoding. This indicates that allocentric viewing preferentially supports the integration of global metric information while reducing reliance on sequential landmark encoding. Regression analyses further showed that landmark sequence knowledge, together with perspective and navigation-duration, uniquely predicted survey knowledge, whereas route metric accuracy did not explain additional variance once these factors were considered.

These results demonstrate that survey knowledge is not a simple accumulation of route-based metric information but results from a selective, context-dependent integration process. The contribution of spatial knowledge varies with environmental structure and perspective: landmarks support the organization of global spatial structure under a ground-level perspective, whereas an aerial perspective prioritizes metric integration over sequential landmark information.

### Future directions and limitations

The present study focused primarily on the behavioral manifestations of spatial knowledge under different navigational conditions. Accordingly, several issues warrant further investigation.

First, the spatiotemporal characteristics of navigation were operationalized through differences in navigation-duration during passive exploration. This approach allowed the study to systematically examine how temporal properties of navigation influence different forms of spatial knowledge. However, the present design did not directly assess participants’ subjective temporal experience during navigation. As a result, interpretations involving temporal distortion or duration-related processing should be understood as theoretically informed accounts that are consistent with the observed behavioral patterns. Future studies may further extend the current framework by incorporating direct measures of subjective temporal experience, thereby clarifying how objective navigation-duration relates to internally experienced navigation time.

Second, the present study mainly examined the behavioral consequences of landmark availability and perspective manipulations, rather than the online cognitive processes through which these factors influence spatial representations. Theoretical interpretations discussed in this study—such as event segmentation, temporal organization, or reference-frame-dependent processing—were derived from existing frameworks in spatial cognition and episodic event research. Although these interpretations are broadly consistent with the current findings, the underlying mechanisms themselves were not directly isolated in the present design. Future work combining behavioral paradigms with process-level measures may help further clarify how landmarks and perspectives shape the temporal and structural organization of navigational experience.

Third, the landmark sequencing task showed relatively limited sensitivity to the experimental manipulations compared with the distance estimation and sketch-map tasks. One possibility is that sequential landmark memory relies on partially different processing strategies from those involved in metric estimation or global structural integration. Another possibility is that participants flexibly shifted between route-based and survey-based representations during task performance. Future studies may benefit from employing more fine-grained measures of sequential spatial knowledge and strategy use to further disentangle these possibilities.

Finally, although the theoretical framework emphasized the joint influence of navigation-duration, landmarks, and perspective, the observed effects were primarily reflected in selective two-way interactions rather than a unified three-way interaction pattern. This suggests that different forms of spatial knowledge may rely on partially distinct processing pathways and information sources. Rather than supporting a single integrated mechanism, the present findings point toward a more flexible and context-dependent account of cognitive map formation. Future research may further explore how these factors interact across different environmental structures, navigation demands, and levels of spatial representation.

## Conclusion

The present study operationalized the spatiotemporal properties of navigation through navigation-duration and examined how navigation-duration, navigational perspective, and landmark availability influenced different forms of spatial knowledge processing during cognitive map formation. The findings revealed clear task-specific and context-dependent effects across tasks. Navigation-duration primarily influenced metric spatial processing, particularly path distance estimation, whereas perspective and landmarks more strongly modulated the organization and integration of spatial information. Different spatial tasks also varied in the extent to which they relied on environmental and navigational information, suggesting that cognitive map formation is not driven by a single source of information but instead emerges from the integration of multiple forms of information encountered during navigation. More importantly, the present findings further indicate that the spatiotemporal properties of navigation may selectively influence metric spatial processing during cognitive map formation.

## Data Availability

The dataset supporting the conclusions of this article is available in the osf.io repository. (https://osf.io/v8m2g/overview?view_only=7042af7096f94eca959388885958f1de).
